# Progranulin: Dose-dependent neurotoxicity

**DOI:** 10.4103/NRR.NRR-D-25-00869

**Published:** 2025-10-30

**Authors:** Shinya Kusakari, Kohsuke Kanekura

**Affiliations:** Department of Pharmacology, Tokyo Medical University, Tokyo, Japan

Progranulin (PGRN), encoded by the *GRN* gene, is a secreted glycoprotein that undergoes proteolytic cleavage to generate individual granulin peptides (granulin A–G) capable of exerting distinct biological functions. PGRN is widely expressed in multiple tissues, including the central nervous and immune systems. Within the central nervous system, PGRN is highly expressed in the hippocampus, cerebral cortex, and hypothalamus, and has been detected in various neuronal subtypes, including Purkinje cells and motor neurons, where it plays a crucial role in neuronal functions, such as neurite outgrowth and synaptic plasticity. In addition to neurons, PGRN is expressed in glial cells, particularly in microglia, where it regulates phagocytosis. Furthermore, PGRN is presented in peripheral immune cells, including macrophages, and contributes to the regulation of inflammatory responses. PGRN exerts its diverse functions via binding partners, including receptors such as sortilin, EphA2, Notch, death receptor 3, and toll-like receptor 9 (Chitramuthu et al., 2017). These interactions underlie the involvement of PGRN in a wide range of cellular processes, including proliferation, differentiation, migration, immune regulation, and tumorigenesis. For instance, the interaction of PGRN with sortilin participates in the endocytosis and lysosomal trafficking of PGRN, whereas Notch signaling influences cell fate decisions and has implications for both neural development and oncogenesis (Chitramuthu et al., 2017).

Given its neurotrophic and anti-inflammatory properties, PGRN is considered a promising therapeutic target for neurodegenerative diseases (Rhinn et al., 2022). Overexpression of PGRN or supplementation with recombinant PGRN has been shown to promote neuronal survival, whereas siRNA-mediated knockdown of PGRN increases vulnerability to apoptosis. Simultaneously, PGRN contributes to lysosomal function by being trafficked to lysosomes via receptors such as sortilin or prosaposin, thereby facilitating lysosomal biogenesis and maintaining lysosomal homeostasis. Disruption of this lysosomal trafficking pathway has been implicated in neuronal storage diseases, highlighting the importance of PGRN in maintaining intracellular homeostasis.

In 2006, *GRN* was identified as the causative gene of frontotemporal dementia (FTD), a neurodegenerative disorder characterized by frontotemporal lobar atrophy, personality changes, and progressive cognitive dysfunction. To date, numerous pathogenic *GRN* mutations have been reported; most of these mutations are nonsense, frameshift, or splice-site mutations that introduce premature stop codons, resulting in nonsense-mediated decay and haploinsufficiency of PGRN (Karamysheva et al., 2019). Consequently, reduced PGRN expression is regarded as a key pathogenic mechanism of FTD. Additionally, several missense mutations in *GRN* have been identified, some of which disrupt protein folding, secretion, and proteolytic processing, further supporting the hypothesis that loss-of-function mutations in the *GRN* gene contribute to disease pathogenesis (Karamysheva et al., 2019).

Corroboratively, PGRN-deficient mice exhibited behavioral abnormalities and neuropathological changes that recapitulate the features observed in patients with FTD. Moreover, *GRN* mutations have been implicated not only in FTD but also in other neurodegenerative diseases such as Alzheimer’s disease, Parkinson’s disease, and amyotrophic lateral sclerosis (Rhinn et al., 2022). Homozygous *GRN* mutations result in a complete PGRN deficiency and cause neuronal ceroid lipofuscinosis type 11 (CLN11), a lysosomal storage disorder characterized by seizures, dementia, and visual impairment. Additionally, non-coding variants of *GRN*, such as rs5848, have been shown to decrease PGRN expression and increase disease susceptibility. Notably, the rs5848 T allele is associated with a higher risk of Alzheimer’s disease and FTD than the C allele. Thus, decreased PGRN expression has been shown to be associated not only with FTD but also with other neurodegenerative diseases. Conversely, it has been suggested that loss of PGRN function alone may be insufficient to cause FTD. In contrast to PGRN homozygous knockout mice, PGRN heterozygous knockout mice do not exhibit age-dependent lipofuscinosis, microglial proliferation, or astroglial proliferation, and neuronal loss and lysosomal pathology are mild. Furthermore, mice with neuron-specific or microglia-specific PGRN deletion do not develop FTD-like pathology.

**Figure 1:**
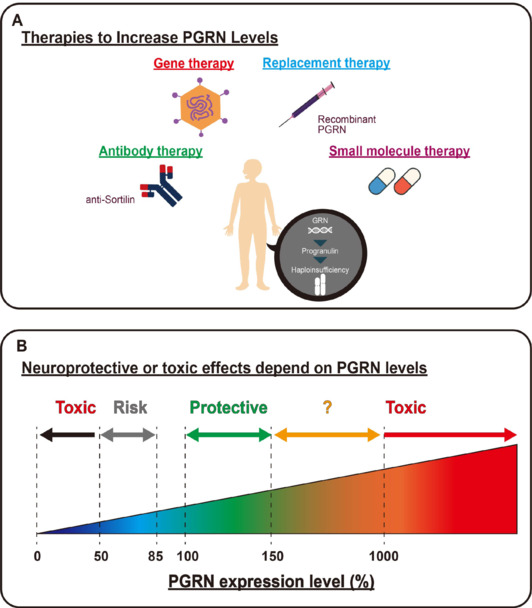
Therapeutic strategies to restore progranulin (PGRN) levels and their potential impact. (A) Schematic overview of therapeutic approaches, including recombinant PGRN administration, antibody therapy (anti-sortilin treatment), gene therapy, replacement therapy, and small molecule therapy under investigation to increase PGRN levels in the context of GRN haploinsufficiency. Adapted from Kusakari et al. (2025). (B) Relationship between PGRN expression levels and their biological effects. While insufficient PGRN is associated with neurodegenerative risk, supraphysiological levels may also induce toxicity. The graph highlights the need to maintain PGRN within an optimal range to achieve neuroprotective effects without adverse effects.

Based on the hypothesis that PGRN replacement may mitigate FTD pathology, several therapeutic approaches, including antibody, gene replacement, and small molecule therapies, are under development (Chitramuthu et al., 2017; Rhinn et al., 2022; **Figure 1A**). Antibody therapy using monoclonal antibodies blocks sortilin, a PGRN receptor involved in controlling extracellular PGRN levels, thereby increasing the circulating PGRN levels. Gene therapy utilizes adeno-associated virus (AAV) vectors to overexpress PGRN by introducing genes into brain cells. In replacement therapy, recombinant PGRN, which increases the efficiency of blood-brain barrier transport, is administered to enhance the amount of circulating PGRN. Therapy using small molecules attempts to increase PGRN protein levels by promoting transcription or inhibiting degradation. These therapeutic approaches are currently being explored in clinical trials and have demonstrated therapeutic effects.

Nevertheless, few studies have explored whether the neurotrophic effect of PGRN is dose-dependent and, in particular, whether high PGRN expression *in vivo* can result in unexpected problems, an essential question in the development of PGRN replacement therapy. Clinical trials on gene therapy using AAV vectors have reported that the levels of neurofilament light chains are elevated after administration (Sevigny et al., 2024). The increased neurofilament light chain levels reflect neurotoxicity, and because the increase in PGRN expression induced by AAV is transient, these results suggest that PGRN overexpression may cause neurotoxicity. Furthermore, analyses using experimental animals have reported the following PGRN-induced toxicities: AAV-mediated PGRN gene delivery to the lateral ventricle caused progressive hippocampal toxicity by leaking PGRN into the cerebrospinal fluid and infiltrating T cells (Amado et al., 2019), and the administration of recombinant PGRN also causes blood–brain barrier impairment (Hummel et al., 2021).

To investigate the safety issues associated with PGRN overexpression *in vivo*, we generated human PGRN transgenic (Tg) mice (Kusakari et al., 2025). In these mice, the CAG promoter drove human PGRN expression at approximately 10-fold higher levels than endogenous mouse PGRN. The Tg mice developed neurodegeneration accompanied by behavioral abnormalities, including a shortened lifespan, age-related neurodegeneration, hyperactivity, cognitive impairment, and motor learning deficits. Histopathological analysis revealed the loss of Purkinje cells in the cerebellum, gliosis (astrocytosis and microgliosis), and lipofuscin accumulation due to lysosomal abnormalities. Interestingly, PGRN Tg mice exhibited a phenotype markedly similar to that of PGRN-deficient mice, suggesting that the overexpression of PGRN may be neurotoxic *in vivo*. Conversely, two Tg mouse lines carrying a single copy of the *GRN* gene have been generated (Beel et al., 2018; Petkau et al., 2021). The Tg mouse established by Petkau et al. (2021) harbors the human *PGRN* gene coding region along with its promoter, inserted into the *Hprt* locus. In contrast, the Tg mouse generated by Beel et al. (2018) carries human PGRN cDNA inserted into the *Rosa* locus. Notably, these mice reportedly do not exhibit neurotoxicity, and PGRN has been shown to exert a protective effect. In both our Tg mouse and these two single-copy Tg mice, exogenous PGRN is expressed in neurons and glial cells in the same pattern as endogenous PGRN. Moreover, PGRN expression paradoxically increases under inflammatory conditions, and it has been suggested that PGRN may exert neurotoxic effects under specific conditions (Philips et al., 2010; Yasui et al., 2011; Tanaka et al., 2022). The observed differences in neurotoxicity are likely attributable to differences in expression levels.

In the present study, we found that overexpression of PGRN using an adenovirus vector induced cell death *in vitro*. PGRN was induced in a dose-dependent manner, and cell death induction by PGRN overexpression required approximately 50-fold higher expression than that of endogenous PGRN in the short term. Furthermore, a more severe phenotype was observed in another founder line with a higher PGRN expression level. Therefore, our results suggest that excessive PGRN expression is cytotoxic, this toxicity is dose-dependent, and that there are specific thresholds for the neuroprotective and neurotoxic effects of PGRN.

Microglia exhibit 50-fold higher PGRN expression than neurons, and deficiency in microglia can result in microglial dysfunction, characterized by impaired phagocytosis, accumulation of myelin debris within microglial lysosomes, an exaggerated inflammatory response following acute injury, and excessive synaptic pruning. These abnormalities suggest that microglial dysfunction plays a key role in the development of FTD associated with *GRN* mutations. However, FTD-like phenotypes have not been observed in microglia-specific PGRN knockout mice.

Interestingly, in our study, astrocytosis was observed across multiple brain regions in PGRN Tg mice, and this condition became more pronounced with age (Kusakari et al., 2025). Conversely, microgliosis appeared to occur transiently, coinciding with the onset of neuronal loss. Furthermore, elevated microglial expression of exogenous PGRN was detected in regions exhibiting severe neurodegeneration, suggesting that microglia may contribute to neurotoxicity induced by PGRN overexpression.

Although many aspects of the mechanism underlying PGRN-induced cytotoxicity remain unclear, it may be explained by several fundamental mechanisms. First, in our *in vitro* experiments, PGRN overexpression was primarily localized to the endoplasmic reticulum (ER), inducing ER stress (Kusakari et al., 2025). Given that chronic ER stress can lead to lysosomal dysfunction, this observation suggests a potential link with the lysosomal abnormalities observed in PGRN Tg mice. However, no clear evidence of ER stress was detected in the brains of Tg mice. In genetically modified animals, adaptive mechanisms may be activated to promote survival. The absence of detectable ER stress induction in PGRN Tg mice may therefore be due to the activation of compensatory mechanisms that suppress ER stress.

Next, PGRN is a secreted protein that undergoes proteolytic processing, a step thought to be essential for regulating its function. A recent study has shown that granulin supplementation has a protective effect in *GRN*-deficient mice (Root et al., 2024). However, proteolytic processing of granulins has also been implicated in the induction of inflammation and cytotoxicity (Horinokita et al., 2019), and the administration of recombinant PGRN causes blood–brain barrier impairment (Hummel et al., 2021). Therefore, excessive increases in granulin may cause neurotoxicity.

In addition, the PGRN expression levels observed in our study greatly exceeded physiological levels. Such excessive increases in expression, whether of PGRN or other proteins, may themselves cause toxicity.

In conclusion, the findings of our study, along with those of several previous reports, suggest that PGRN does not inherently exert neuroprotective effects under all conditions. Notably, PGRN exhibits dual properties, exerting neuroprotective effects at physiological levels and inducing neurotoxic effects when overexpressed (**Figure 1B**). These findings highlight critical considerations in the development of PGRN-targeted therapies: treatment strategies should aim to restore PGRN levels to physiological ranges rather than simply promoting their overexpression.

Recent studies in mice have shown that supplementation with PGRN or granulins via AAV delivery can suppress FTD-like symptoms caused by PGRN deficiency without inducing toxicity (Reich et al., 2024; Root et al., 2024). Therefore, careful adjustment of PGRN expression levels is essential. Future studies should investigate the dose-response relationships across different cell types, control expression using tissue-specific promoters, and monitor granulin fragment profiles to assess potential toxicity. Refining the pattern and extent of PGRN enhancement is key to maximizing therapeutic efficacy while minimizing side effects.

*We thank Hiroaki Suzuki and Mikiro Nawa for their insightful discussions and valuable suggestions during the preparation of this perspective, and Tomoko Ohara, Yuka Toyama, and Takako Hiraki (Tokyo Medical University, Japan) for their valuable assistance.*

*This work was supported by SENSHIN Medical Research Foundation, Takeda Science Foundation, Taiju Life Social Welfare Foundation, Mitsui Sumitomo Insurance Welfare Foundation, Research Foundation for Pharmaceutical Sciences, Tokyo Medical University Research Grant, JSPS KAKENHI (23K06369) to SK, and JSPS KAKENHI (24K02187) to KK.*

**Additional file:** *Open peer review report 1.*

OPEN PEER REVIEW REPORT 1
