# Supplementary material for: Progranulin: Dose-dependent neurotoxicity
Source: Neural Regen Res. 2025 Oct 30;21(7):2958–9. doi: 10.4103/NRR.NRR-D-25-00869 (PMC13378914; doi:10.4103/NRR.NRR-D-25-00869)
Supplement: Supplementary file 1 [file NRR-21-2958_Suppl1.pdf]

OPEN PEER REVIEW REPORT 1

Name of journal: Neural Regeneration Research
Manuscript NO: NRR-D-25-00869
Title: Dose-dependent neurotoxicity of progranulin
Reviewer's Name: Thomas Kukar
Reviewer's country: USA

COMMENTS TO AUTHORS

This interesting perspective article follows a recent article from the authors where they describe transgenic mouse and cell models that massively over express human Progranulin leading to toxicity. The take home message that "careful adjustment of PGRN expression levels is essential" for "maximizing therapeutic efficacy while minimizing side effects" is well supported by the authors' studies and the broader PGRN literature. However, there are several issues to be addressed that would strengthen the perspective and be more accurate and aligned with recent PGRN studies.

* The statement that PGRN functions as "an autocrine and paracrine growth factor" is not accurate. It certainly has been suggested that it could function in that way. But the literature is replete with examples that PGRN may NOT function in the manner.
* For example, the idea that PGRN interacts with TNFR and modulates inflammatory cascades has not been widely replicated and most in the field do not think PGRN directly alters TNF signaling (see PMIDs: 23699531, 24100384, 29740434).
* It is important to acknowledge that the described human PGRN Tg mice express supraphysiological levels of PGRN. In fact, the original paper suggests 18 copies of the transgene were inserted into the mouse genome. There are many reasons that high level, unregulated expression of many proteins could cause toxicity in mice.
* Do these mice also express normal, endogenous mouse PGRN? If so, this further confounds interpretation of the results.
* It is unclear what the following statement means "the cytotoxic effects of abnormal PGRN species may also contribute to the pathogenesis of FTD"? Based on what evidence?
* Furthermore, the statement "resulting granulin cleavage products of PGRN have been implicated in modulating inflammation and inducing cytotoxicity" is inaccurate. In fact, a recent study finds that cleaved granulin products fully rescue PGRN-deficiency, with no evidence of toxicity (PMID: 39565694).
* Finally, there are multiple additional studies using AAV to express PGRN without overt toxicity (PMID: 38838131).
